# Seroprevalence, spatial distribution, and social determinants of SARS-CoV-2 in three urban centers of Chile

**DOI:** 10.1186/s12879-022-07045-7

**Published:** 2022-01-28

**Authors:** Pablo Vial, Claudia González, Gloria Icaza, Muriel Ramirez-Santana, Rubén Quezada-Gaete, Loreto Núñez-Franz, Mauricio Apablaza, Cecilia Vial, Paola Rubilar, Juan Correa, Claudia Pérez, Andrei Florea, Eugenio Guzmán, María-Estela Lavín, Paula Concha, Manuel Nájera, Ximena Aguilera

**Affiliations:** 1 Instituto de Ciencias e Innovación en Medicina, Facultad de Medicina Clínica Alemana, Universidad del Desarrollo, Av. Plaza #680, San Carlos de Apoquindo, 7610658 Las Condes, Santiago Chile; 2 Centro de Epidemiología y Políticas de Salud, Facultad de Medicina Clínica Alemana Universidad del Desarrollo, Av. Plaza #680, San Carlos de Apoquindo, 7610658 Las Condes, Santiago Chile; 3 Instituto de Matemáticas, Universidad de Talca, Avenida Uno Poniente #1141, 3460000 Talca, Chile; 4 Public Health Department, Faculty of Medicine, Universidad Católica del Norte, Larrondo 1281, 1780000 Coquimbo, Chile; 5 Departamento de Salud Pública, Facultad de Ciencias de la Salud, Universidad de Talca, Avenida Uno Poniente #1141, 3460000 Talca, Chile; 6 Facultad de Gobierno, Universidad del Desarrollo, Av. Plaza #680, San Carlos de Apoquindo, 7610658 Las Condes, Santiago Chile; 7 Centro Producción del Espacio, Universidad de Las Américas, Avenida Manuel Montt #948, 7500975 Providencia, Santiago Chile; 8 Escuela de Enfermería, Facultad de Medicina Clínica Alemana Universidad del Desarrollo, Av. Plaza #680, San Carlos de Apoquindo, 7610658 Las Condes, Santiago Chile

**Keywords:** COVID-19, Seroprevalence, Spatial analysis, Chile, Case fatality ratio, Attack rate, Secondary attack rate, Geography, Household transmission

## Abstract

**Background:**

Seroprevalence studies provide an accurate measure of SARS-CoV-2 spread and the presence of asymptomatic cases. They also provide information on the uneven impact of the pandemic, pointing out vulnerable groups to prioritize which is particularly relevant in unequal societies. However, due to their high cost, they provide limited evidence of spatial spread of the pandemic specially in unequal societies. Our objective was to estimate the prevalence of SARS-CoV-2 antibodies in Chile and model its spatial risk distribution.

**Methods:**

During Oct–Nov 2020, we conducted a population-based serosurvey in Santiago, Talca, and Coquimbo–La Serena (2493 individuals). We explored the individual association between positive results and socio-economic and health-related variables by logistic regression for complex surveys. Then, using an Empirical Bayesian Kriging model, we estimated the infection risk spatial distribution using individual and census information, and compared these results with official records.

**Results:**

Seroprevalence was 10.4% (95% CI 7.8–13.7%), ranging from 2% (Talca) to 11% (Santiago), almost three times the number officially reported. Approximately 36% of these were asymptomatic, reaching 82% below 15 years old. Seroprevalence was associated with the city of residence, previous COVID-19 diagnosis, contact with confirmed cases (especially at household), and foreign nationality. The spatial model accurately interpolated the distribution of disease risk within the cities finding significant differences in the predicted probabilities of SARS-CoV-2 infection by census zone (IQR 2.5–15.0%), related to population density and education.

**Conclusions:**

Our results underscore the transmission heterogeneity of SARS-CoV-2 within and across three urban centers of Chile. Socio-economic factors and the outcomes of this seroprevalence study enable us to identify priority areas for intervention. Our methodological approach and results can help guide the design of interdisciplinary strategies for urban contexts, not only for SARS-CoV-2 but also for other communicable diseases.

**Supplementary Information:**

The online version contains supplementary material available at 10.1186/s12879-022-07045-7.

## Background

After emerging in Asia, the first wave of COVID-19 took place in the southern hemisphere between March and October 2020 [1]. The first Chilean COVID-19 case was identified on March 3, 2020, becoming by mid-June 2020 one the world’s most per capita affected countries, centered mainly in the capital, Santiago, which accounts for 40% of the country's population [2, 3]. Due to Chilean geography, climate heterogeneity, and other characteristics, each province has presented its own epidemic curve [3].

The SARS-CoV-2 seroprevalence outcomes vary substantially from place to place. Aside from the timeframe, the disease incidence not only depends on the public health response in each country, but also on spatial distribution, population density, and physical layouts of cities where the pathogen is introduced [4].

It has been well established that social, economic, and cultural forces shape the spread and impact of infectious diseases in human populations [5]. Recent studies provided evidence on the relationship between social status and exposure patterns, the likelihood of diagnosis, and infection outcomes for COVID-19 in Santiago. But these studies have an ecological design and are based on administrative registries of morbidity or mortality, and have ignored the effect of social determinants in other urban centers of Chile [6–8].

Epidemiological surveillance systems for SARS-CoV-2 epidemic have focused, primarily on registries of symptomatic and severe cases and deaths, based on molecular testing capacities using polymerase chain reaction (Rt-PCR). Seroprevalence studies, using antibodies detection, have identified the presence of mild and asymptomatic SARS-CoV-2 infections not registered by surveillance systems relying on symptoms [9–13]. Thus, seroprevalence studies identify the infection burden in communities leading to more appropriate control measures for local epidemic expansion. They provide information quantifying the susceptibility and the immunity status of the participants, inferring the dynamics epidemic infection in a population. At the same time, these studies may highlight risk factors for infection, severity, and lethality [9, 14, 15] .

The seroprevalence during the first wave in Europe ranged from 0.42% among residential clinical samples in Greece, to 23% in Lodi (blood donors), and 23.6% in Castiglione D'Adda (population based), both located in highly affected places in north of Italy [10, 16, 17]. Latin America was severely affected, with the world’s highest per capita death toll rate in Perú and the third largest toll of cases in Brazil [18], evidencing disparity in pandemic consequences in a deeply unequal sub-continent [19]. Nevertheless, few studies report the seroprevalence in Latin America with a range from 8.3% to 44% in diverse localities [9, 20–22].

Until this study, there have been no reports of population-based seroprevalence studies in Chile. This article reports a population-based seroprevalence study conducted in three urban centers located in the center-north, metropolitan, and center-south regions of Chile: Coquimbo-La Serena conurbation, Santiago (capital city), and Talca. Using geospatial modelling, the distribution of SARS-CoV-2 infection risk was identified, based on individual and ecological data, in these three locations.

## Methods

### Study design and participants

A cross-sectional population-based survey was conducted to measure the seroprevalence of SARS-CoV-2 antibodies in residents (seven years and older) of three urban areas in the center zone of Chile: Greater Santiago (7,000,000 inhabitants), Coquimbo-La Serena conurbation (nearly 500,000 inhabitants), and Talca (over 200,000 inhabitants) [23]. Fieldwork was carried out from September 25–November 25, 2020, after the country's first epidemic wave.

Participants were selected using two-stage stratified sampling. For Coquimbo- La Serena and Talca, the first level of stratification was census tracts; the number of census tracts for both cities are 19 and 15, respectively [23]. The sample was distributed proportionally to the number of dwellings in each census tract. Subsequently, blocks with dwellings were listed and randomly selected. Finally, dwellings were systematically selected and inquired to participate in the study.

In Santiago, we used a panel sample from the School of Government of the Universidad del Desarrollo surveys, that considered 34 urban municipalities as the first stratification level. The sample was distributed proportionally to the number of dwellings in each municipality. More detail in Additional file 1, page 1:Sample estimation for Santiago.

Sample size assumed 7% infection prevalence, with a precision of ± 3%, a confidence level of 95%, with a design effect of 2, obtaining a minimum necessary size of 556. In Santiago, we doubled this number. We estimated the number of dwellings using the observed average persons per dwelling (2.7 in Santiago, 3.04 in Coquimbo-La Serena, and 2.93 in Talca) [23].

Consenting participants completed an on-site questionnaire requesting independent variables: basic demographics (age, sex, nationality); socio-economics (education, ethnic background, health insurance); housing conditions (type of dwelling, overcrowding, heating fuel); work-related (health care workers, face-to-face work); COVID-19 exposure (contact with confirmed cases and places of contact); self-reported COVID-19 diagnosis; COVID-19 related symptoms, and comorbidities, including tobacco consumption. Health insurance considered public, private, armed forces or other, and overcrowding was defined as 2.5 individuals or more per bedroom.

Data were collected and managed using REDCap® electronic data capture tools hosted at Universidad del Desarrollo.

Ecological data was gathered from the National Census [23], at the census zone scale, including population density (person/dwelling), number of households per dwellings; overcrowding; housing sanitation (tap water/sewage coverage); masculinity index; nationality composition; unemployment rate; and level of education.

Data on morbidity and cause of death were captured from the Chilean Ministry of Health administrative data (MOH) [3, 24]. The selected death codes from the ICD-10 were: U07.1 (COVID-19, virus identified) and U07.2 (COVID-19, virus not identified).

### Laboratory methods

All participants underwent venipuncture on-site. Serum was separated within 24 h in local laboratories, aliquoted, and shipped at 4 ºC to the Clinica Alemana de Santiago laboratory for processing. Detection of SARS-CoV-2 antibodies was carried out using the immunoassay Elecsys Anti-SARS-CoV-2 (ELISA) (Roche® with a cobas® analyzer series). The technique is a Pan-Ig, detects indistinctly IgM and IgG antibodies, the first pointed out acute response, while the second indicates a more prolonged serological response.

The manufacturer’s reports indicated clinical sensitivity 14 days post PCR confirmation of 99% (95% CI 97.0–100%); analytical specificity of 100%, and clinical specificity of 99.8% (95% CI 99.7–99.9%) [25].

A point-of-care rapid test (immunochromatographic assays for IgG detection Zhuhai Livzon®) was used in case venipuncture failed or for a contraindication to test, or for young children who refused venipuncture. Manufacturer reports indicate sensitivity 90.6% and specificity 99.2%.

### Statistical analysis

The dependent variable, is the presence of SARS-CoV-2 antibodies (ELISA), or IgG (rapid test), estimated the seroprevalence of COVID-19 as the proportion of seropositive individuals, adjusted by sampling weights (Additional file 1, page 2: Sample weights calculation). Seroprevalence estimates were stratified by independent variables and proportion of asymptomatic cases among seropositive individuals was also estimated.

The Infection Fatality Ratio (IFR) was calculated using officially reported number of deaths as the numerator with our estimates of total number of infected people for each city as the denominator.

The association between the presence of antibodies against SARS-CoV-2 and each independent variable was estimated using logistic regression from complex surveys. Secondly, a multivariate logistic regression from complex surveys analysis was implemented: First, all socio-economic and ecological variables described above were included. Then, non-significant independent variables (p > 0.0), except age and sex, were systematically excluded to obtain a parsimonious model explaining the risk positive results. In addition, the concordance index (c-index) was used to select the final model. Finally, probabilities at the individual level were estimated and spatially plotted, ensuring the anonymity of households and individuals.

Statistical analyses were carried out using SAS and Stata (version 9. and 15 respectively).

### Geographical modelling

A spatial interpolation was done using Empirical Bayesian Kriging (EBK) for each city. This is an adaptive interpolation model to generate estimates of the distribution of a certain variable in space, from the observed distribution of the points of that variable in space [26–29]. The interpolation was based on the variables resulting from the multivariate analysis of the individual seroprevalence data and ecological variables (with census zone and municipal level scales). EBK used individual geolocated data through the application of a standard circular four section in 45 degrees. The sampling distance (radius) estimated by EBK was: 400 m in Talca, and 600 m in Santiago and Coquimbo-La Serena. The number of neighbors ranged from 10 to 15, with a semivariogram power and 100 simulations (more detail in Additional file 1, page 4: Empirical Bayesian Kriging).

Finally, we confronted our results by performing a correlation analysis of our spatial model results compared to the official surveillance data provided by the MOH. These data comprise the number of COVID-19 confirmed cases from the first case until ending fieldwork (epidemiological week 48, 2020) at the census zone scale.

Geographical analysis we used ArcGis 10.7.

The Ethics Committees of the Universities el Desarrollo and Talca and the Faculty of Medicine of the Universidad Catolica del Norte (Numbers 2020-54, 34-2020, and 21-2020, respectively) approved the study protocols.

Informed consent was obtained from all subjects, if subjects are under 18, from a parent and/or legal guardian. All methods were carried out in accordance with relevant guidelines and regulations in ethical approval and consent to participate section.

### Role of funding source

The founder did not have any role in design, analysis, interpretation or writing of the reports.

## Results

**Fig. 1:**
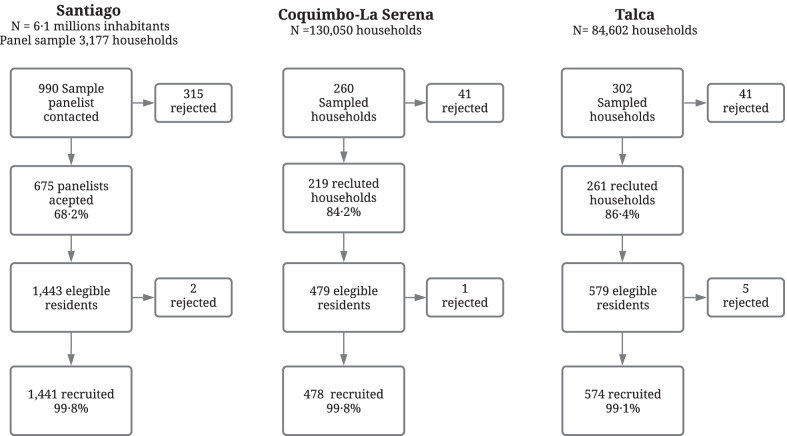
Flowchart participants in seroprevalence of SARS-CoV-2 antibodies study in three urban centers of Chile 2020

We enrolled 1155 dwellings; refusal to participate was 25.6% at the first contact and less than 1% among residents of the recruited dwellings (Fig. 1). A total of 2493 subjects were enrolled, between 7 and 94 years old (average 41.6 years); 60.3% female; 1441 were residents of Santiago (57.8%); 478 from Coquimbo-La Serena (19.2%) and 574 from Talca (23.0%) (Additional file 1, page 3: Table S1). The ELISA test was performed in 95.2% of the participants; this proportion was lower in Talca (90.1%). All point-of-care tests were negative (n = 120).

**Fig. 2:**
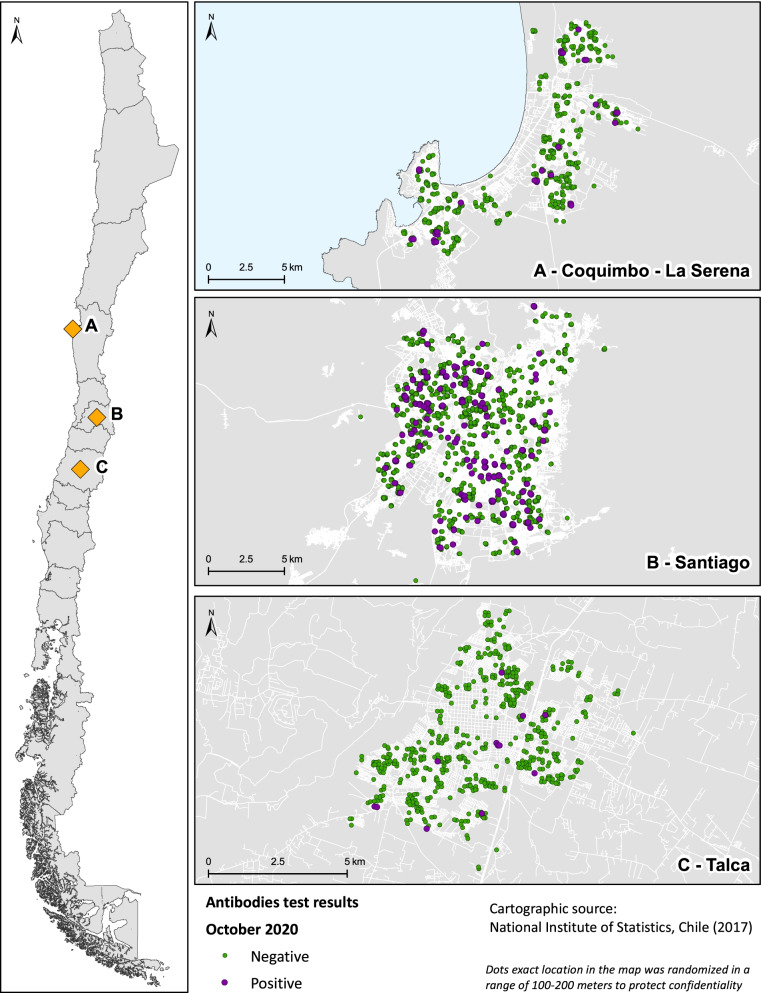
Geographical distribution of study sites and SARS-CoV-2 antibodies in three urban centers of Chile 2020

Between September 26 and November 25, 2020, the population seroprevalence was significantly higher in Santiago (11.0%, 95% CI 8.2–14.7), followed by Coquimbo-La Serena (5.6%, 95% CI 3.3–9.5) and lower in Talca 2.0% (95% CI 0.8–4.7). Figure 2 shows the geographical distribution of study sites and antibodies results.

The overall prevalence was 10.4% (95% CI 7.8–13.7), representing 734,220 individuals who have been infected with SARS-CoV-2 (Table 1). We found no differences in seroprevalence according to sex, age, or ethnicity. However, Coquimbo-La Serena indicated a significant increase with age up to 59 years (p = 0.0263) (Additional file 1, page 5:Table S2). Regarding nationality, foreigners had higher seroprevalence, reaching statistical significance only in Talca (p = 0.0253).

**Table 1:** Population seroprevalence of SARS-CoV-2 and sociodemographic variables, Chile 2020

	Participants (n)	Seropositive (n)	Seroprevalence^a^ % (95% CI)	OR (95% CI)
Overall	2493	242	10.4 (7.8–13.7)	–
City:				
-Coquimbo- La Serena	478	30	5.6 (3.3–9.5)	2.91 (1.01–8.44)
-Santiago	1441	200	11.0 (8.2–14.7)	6.1 (2.3–15.8)
-Talca	574	12	2.0 (0.8–4.7)	Ref
Sex:				
-Male	990	107	12.2 (8.3–17.5)	1.5 (0.9–2.4)
-Female	1503	135	8.7 (6.2–12.0)	Ref
Age Group:				
-7–14	176	21	12.5 (6.1–23.8)	Ref
-15–24	360	38	9.0 (5.2–15.3)	0.70 (0.26–1.86)
-25–39	649	62	10.4 (6.5–16.2)	0.81 (0.32–2.04)
-40–59	813	93	12.1 (7.8–18.3)	0.97 (0.42–2.22)
-≥ 60	495	28	6.7 (3.4–12.9)	0.50 (0.18–1.41)
Nationality:				
-Chilean	2378	221	9.3 (7.1–12.1)	Ref
-Foreign	115	21	21.6 (9.1–43.2)	2.69 (0.96–7.56)
Native South American Ethnicity:^b^				
-Yes	230	23	17.8 (7.0–38.6)	2.0 (0.66–6.03)
-No	2263	219	9.7 (7.3–13.0)	Ref
Educational level (for participants > 17 years old) (n = 2234):				
-None or primary	234	11	4.3 (1.6–11.2)	0.63 (0.19–2.09)
-Secondary	1130	130	14.2 (10.0–19.8)	2.30 (1.16–4.54)
-Technical	330	38	9.5 (5.1–17.0)	1.46 (0.59–3.57)
-University	540	36	6.7 (3.7–11.9)	Ref
Health Insurance (n = 2347):				
-Public	1811	192	12.7 (9.3–17.2)	2.5 (1.25–5.10)
-Private	536	44	5.5 (2.9–9.9)	Ref
Overcrowding (n = 2487):				
-No	2338	219	10.7 (7.9–14.2)	Ref
-Yes	149	23	7.5 (2.9–18.1)	0.68 (0.24–1.96)
Number of residents:				
-< 5	1651	9.3	6.2–13.8	Ref
-≥ 5	842	12.8	8.9–18.2	1.43 (0.79–2.62)
Type of dwelling (n = 2486):				
-House	2142	201	10.3 (7.7–13.6)	Ref
-Apartment	344	38	10.5 (6.1–17.5)	1.02 (0.52–2.02)
Heating Fuel (2486) Gas:				
-No	1315	120	9.4 (6.6–13.2)	Ref
-Yes	1178	122	11.4 (7.4–17.2)	1.24 (0.67–2.30)
Kerosene:				
-No	1907	181	10.5 (7.4–14.5)	Ref
-Yes	586	61	10.2 (6.0–16.7)	0.97 (0.49–1.92)
Firewood:				
-No	2175	229	10.5 (7.8–13.9)	Ref
-Yes	318	13	7.1 (3.4–14.5)	0.66 (0.28–1.54)
Electricity:				
-No	1647	159	11.5 (7.9–16.4)	Ref
-Yes	846	83	8.7 (5.6–13.2)	0.73 (0.39–1.36)

OR, odds ratio; ref, reference category; 95% CI, 95% confidence interval. ^a^Weighted for sampling weights. ^b^By self-identification

In terms of socio-economic risk factors (Table 1), people with public health insurance showed a higher seroprevalence, both in Santiago and in the pooled results (OR = 2.5, 95% CI 1.3–5.1). Likewise, a significant association with educational level was observed: people with university education had a lower seroprevalence than those who completed secondary education (OR = 2.3, 95% CI 1.2–4. 5). However, no association was found between seroprevalence and other social-related variables at the individual level, such as overcrowding, number of residents, or other housing conditions. Neither was an association found between the type of occupation or face-to-face work with the presence of antibodies.

Most seropositive cases were symptomatic (153/242; 63. 2%), and presence of symptoms was strongly associated with seropositivity (OR = 5.6, 95% CI 3.3–9.4) (Table 2 and Additional file 1, page 6: Table S3). Likewise, the number of symptoms positively associated with seroprevalence (Mantel–Haenszel = 296; p < 0.0001). Only 38.7% (252/651) of people with symptoms sought medical care. Asymptomatic infection was more frequent in children below 15 years old (p = 0.0304).

**Table 2:** Seroprevalence of SARS-CoV-2 according to contact history and clinical characteristics

	Participants (n = 2493)	Seropositive participants (n = 242)	Seroprevalence^a^ % (95% CI)	OR (95% CI)
COVID-19 diagnosis (n = 2493):				
-No	2373	144	6.4 (4.7–8.8)	Ref
-Yes	120	98	68.8 (43.5–86.3)	32.0 (10.7–96.3)
COVID-19 hospitalization (n = 120):				
-No	109	88	69.5 (50.8–83.4)	Ref
-Yes	11	10	59.3 (43.7–73.3)	0.6 (0.2–1.8)
Contact with confirmed cases (n = 2492):				
-No	2196	135	7.4 (5.3–10.2)	Ref
-Yes (≥ 1 person)	296	107	31.2 (20.2–44.8)	5.7 (3.0–10.9)
Exposure site (n = 296):				
-At home	197	82	42.1 (21.1–58.7)	3.9 (1.1–13.2)
-Work/small gatherings/others	99	25	15.8 (6.3–34.5)	Ref
Quarantine (n = 296):				
-No	123	32	21.9 (10.9–39.1)	Ref
-Yes	173	75	41.0 (24.9–59.3)	2.5 (0.9–7.3)
Any symptom:^b^				
-No	1841	89	5.2 (3.5–7.7)	Ref
-Yes	652	153	23.6 (17.1–31.6)	5.6 (3.03–9.4)
Number of symptoms:				
-None	1841	89	5.2 (3.5–7.7)	Ref
-1–2 symptoms	275	32	19.1 (9.8–33.9)	4.3 (1.8–10.0)
-2–3 symptoms	195	46	19.6 (11.5–31.2)	4.4 (2.1–9.2)
-≥ 5 symptoms	182	75	38.1 (24.6–53.8)	11.2 (5.6–22.3)
Seek medical care (n = 651):				
-No	399	64	17.7 (11.5–26.1)	Ref
-Yes	252	89	32.2 (21.5–45.1)	2.21 (1.1–4.4)

Chile 2020

OR, odds ratio; ref, reference category; 95% CI, 95% confidence interval. ^a^Weighted for sampling weights. ^b^COVID-19 compatible symptoms including fever, cough, odynophagia, dyspnea, headache, myalgia, chest pain, abdominal pain, diarrhea, fatigue, anosmia and dysgeusia

The most frequent symptoms in seropositive subjects were: headache, myalgia, and odynophagia. In contrast, the greatest strength of association with antibodies presence was for anosmia and dysgeusia (OR = 30.0, 95% CI 12.1–74.6 and OR = 32.9, 95% CI 11.6–93.9 respectively) (Table 3 and Additional file 1, page 7: Table S4). No association was observed between seroprevalence and self-reported chronic conditions, except for tobacco consumption, which appears to have a negative association with infection (OR = 0.5, 95% CI 0.3–0.9) (Additional file 1, pages 8-9: Tables S5 and S6).

**Table 3:** Frequency and type of symptoms and presence of SARS-CoV-2 antibodies. Chile 2020

Symptoms	Participants			Symptoms in seropositive^a^ (%)*	Symptoms in seronegative^a^ (%)*	OR (95% CI)^b^
	Total (n = 2493)	Seropositive (n)	Seronegative (n)			
Fever	173	65	108	24.9	4.8	6.6 (3.4–12.8)
Cough	237	63	174	27	8.2	4.1 (2.0–8.8)
Odynophagia	326	75	251	29.5	12.1	3.0 (1.7–5.4)
Dyspnea	147	49	98	15	4.6	3.7 (1.8–7.4)
Headache	434	113	321	45.9	15.9	4.5 (2.5–8.2)
Myalgia	306	94	212	39.4	10.7	5.4 (3.1–9.5)
Chest pain	108	45	63	22.2	3.2	8.8 (3.4–22.9)
Abdominal pain/diarrhea	168	50	118	19.2	7.5	3.0 (1.3–6.5)
Fatigue/prostration	181	61	120	24.9	5.5	5.7 (3.0–10.9)
Anosmia	100	74	26	23.3	1.0	30.0 (12.1–74.6)
Dysgeusia	89	66	23	21.6	0.8	32.9 (11.6–93.9)

OR, odds ratio; 95% CI, 95% confidence interval. ^a^Weighted for sampling weights. ^b^Reference category = seronegative

Previous COVID-19 diagnosis was reported by 120 subjects, with 81.7% seropositivity. This antecedent was strongly associated with seropositivity (overall OR = 32.0, 95% CI 10.7–96.3). The hospitalization rate for self-reported COVID-19 subjects was 10% (Table 2). Seroprevalence was also significantly higher in those who reported contact with confirmed cases (31.2% OR = 5.7 95% CI 3.0–10.9). The most frequent contact place was the home, with higher seroprevalence than other contact places (42.1%, OR = 3.9 95% CI 1.1–13.3). More than half of those with close contact confirmed cases were quarantined (173/296; 58.4%); this proportion was higher in Coquimbo-La Serena (63.3%). Coquimbo-La Serena was the only city where quarantine history was associated with seropositivity (p < 0.0001) (Table 2 and Additional file 1, page 6:Table S3). Two COVID-19 related deaths occurred among the residents of the sampled dwellings, both males over 60 years residents of Santiago.

Table 4 shows the estimation of the infection fatality ratio (IFR) for the three cities, the average risk of death for SARS-CoV-2 infected people was 1.3% (95% CI 1.27–1.32) for PCR confirmed deaths and increased to 1.67% (95% CI 1.64–1.70) when considering all COVID-19 attributable deaths.

**Table 4:** Estimation of SARS-CoV-2 infection fatality ratio from seroprevalence, Chile 2020

	Registered deaths 7 years and older	Population 7 years and older	Seroprevalence% (95% CI)	Estimate number of infected cases	Infection Fatality Ratio	
					%	95% CI^c^
Coquimbo-La Serena	–	459,950	5.61 (3.3–9.5)	25,803	–	–
COVID-19 confirmed deaths^a^	152	–	–	–	0.59	(0.50–0.69)
COVID-19 attributed deaths^b^	218	–	–	–	0.84	(0.74–0.96)
Santiago	–	6,382,709	11.03 (8.2–14.7)	704,013	–	–
COVID-19 confirmed deaths^a^	9279	–	–	–	1.32	(1.29–1.35)
COVID-19 attributed deaths^b^	11,922	–	–	–	1.69	(1.66–1.72)
Talca	–	217,147	2.00 (0.8–4.7)	4343	–	.
COVID-19 confirmed deaths^a^	112	–	–	–	2.58	(2.15–3.09)
COVID-19 attributed deaths^b^	132	–	–	–	3.04	(2.57–3.59)
Overall	–	7,059,806	10.40 (7.8–13.7)	734,159	–	–
COVID-19 confirmed deaths^a^	9543	–	–	–	1.30	(1.27–1.32)
COVID-19 attributed deaths^b^	12,272	–	–	–	1.67	(1.64–1.70)

95% CI, 95% confidence interval. ^a^International Classification of diseases (ICD-10) U07.1. ^b^ICD-10 U07.1 and U07.2. ^c^Wilson score

The proportion of dwellings with at least one seropositive resident reached 12.8% and was higher in Santiago (Table 5). In those dwellings, the average of infected residents was 61.6% (95% CI 56.6–66.4), and the secondary attack rate reached 38.4% (95% CI 32.2–44.8%).

**Table 5:** SARS-CoV-2 seroprevalence by dwelling and secondary attack rate

	Dwellings	Dwellings with seropositive residents		Residents^a^	Seropositive residents		2nd attack rate^c^
		n	% (95%CI)	n	n	Attack rate%^b^ (95% CI)	% (95% CI)
Coquimbo-La Serena	219	16	7.3% (4.2–11.5)	46	30	65.2% (49.8–78.6)	46.7% (28.3–65.7)
Santiago	675	123	18.2% (15.4–21.3)	317	200	63.1% (57.5–68.4)	39.7% (32.7- 46.9)
Talca	261	9	3.5% (1.6–6.4)	30	12	40.0% (22.7–59.4)	14.3% (3.0–36.3)
Total	1155	148	12.8% (10.9–14.9)	393	242	61.6% (56.6–66.4)	38.4% (32.2–44.8)

Chile 2020

95% CI, 95% confidence interval. ^a^no of people in dwellings with a seropositive member. ^b^(seropositive residents/total residents). ^c^(seropositive residents less 1 seropositive per dwelling)/(total residents less 1 seropositive per dwelling)

Table 6 shows the bivariate analysis between the presence of SARS-CoV-2 antibodies and ecological variables. Having antibodies against SARS-CoV-2 is significantly associated with high population density (p = 0.004) and with a higher proportion of individuals without university education (p = 0.005).

**Table 6:** Social variables according to SARS-CoV-2 antibodies results by census zone

	Seropositive mean/SD	Seronegative mean/SD	*P* value (two sided)
% Individuals without access to tap water	1.5%/0.02	1.6%/0.01	0.865
% Dwellings with more than one household	2.1%/0.2	1.9%/0.07	0.394
% Dwellings with overcrowding^a^	7.6%/0.6	6.7%/0.3	0.202
% Individuals without university education	76.4%/2.9	64.5%/1.6	0.005
% Migrant population	7.3%/1.0	9.5%/0.6	0.17
% Unemployed population	6.8%/0.2	6.4%/0.1	0.149
% Males	48.1%/0.2	48.0%/0.09	0.648
Population density^b^	3.1/6.4	2.8/3.9	0.004

Chile 2020

SD, standard deviation. ^a^Overcrowding ≥ 2.5 people/bedroom ^b^Population density = people/dwellings

Multivariate model shows that at the individual level, the risk factors of SARS-CoV-2 infection are contact with confirmed cases and foreign nationality. At the ecological level, a positive association exists between seropositivity, population density, and the proportion of individuals with university education. When comparing the three cities, this model shows significant differences in the risk of SARS-CoV-2 infection only between Santiago and Talca. The selected multivariate logistic model reached a concordance index of 0.80. Final results are provided in Table 7.

**Table 7:** Multivariate model of individual and ecological risk factors associated with SARS-CoV-2 antibodies

Category	Multivariable OR^a^	95% CI	*P* value (two sided)
City:			
-Talca	Ref	–	–
-Coquimbo-La Serena	2.6	0.9–7.4	0.0795
-Santiago	4.8	1.9–12.6	0.0013
Contact with a confirmed case:			
-No	Ref	–	–
-Yes	4.2	2.1–8.2	< 0.0001
Foreign:			
-No	Ref	–	–
-Yes	4.7	1.8–12.7	0.0020
Sex:			
-Female	Ref	–	–
-Male	1.5	0.9–2.5	0.0966
Age:			
-7–14	Ref	–	–
-15–24	0.9	0.4–2.3	0.8967
-25–39	1.1	0.5–2.5	0.7904
-40–59	1.1	0.5–2.3	0.7720
-60 and more	1.0	0.4–2.7	0.9858
People without university education (%)^b^	4.0	1.2–14.1	0.0296
Population density^b^	1.8	1.0–3.0	0.0387

OR, odds ratio; 95% CI, 95% confidence interval. ^a^Weighted for sampling weights. ^b^Per census zone

The spatial EBK interpolation model using the multivariate prediction (education and population density at the census zone) together with seroprevalence results was representative for the three cities (root mean square standardized error > 0.9). The range of predicted probabilities of SARS-CoV-2 infection were 0.008–0.756, 0.006–0.497, and 0.002–0.265 in Santiago, Coquimbo-La Serena, and Talca, respectively.

**Fig. 3:**
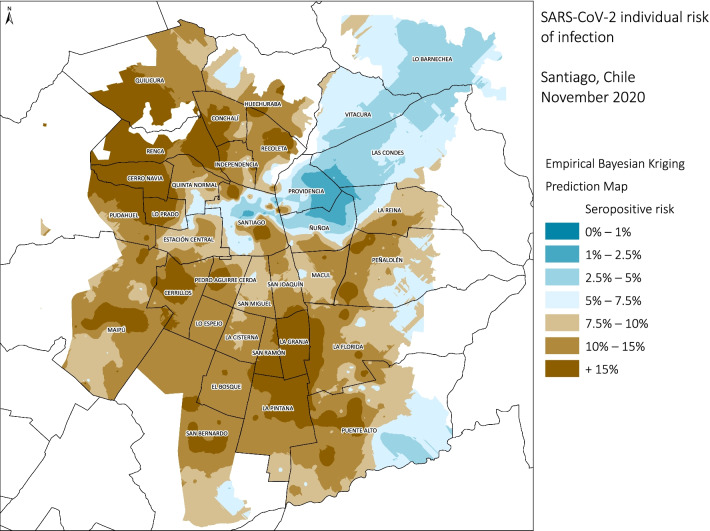
Empirical Bayesian Kriging predicted values for SARS-CoV-2 individual risk of infection for Santiago, Chile 2020

**Fig. 4:**
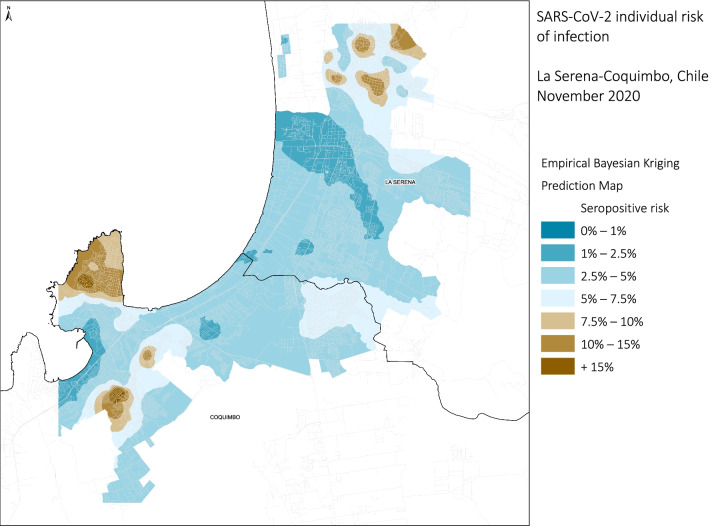
Empirical Bayesian Kriging, predicted values for SARS-CoV-2 individual risk of infection for La Serena-Coquimbo, Chile. 2020

**Fig. 5:**
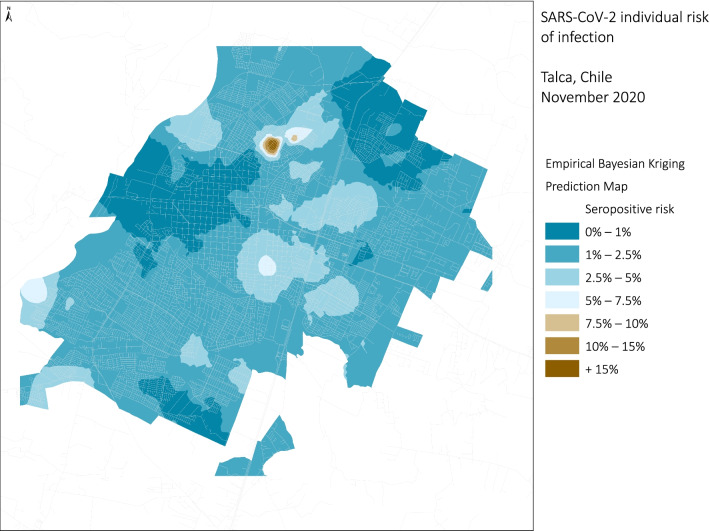
Empirical Bayesian Kriging, predicted values for SARS-CoV-2 individual risk of infection for Talca, Chile 2020

In Santiago (Fig. 3), the territorial distribution of the estimated model by census zone showed that in a large part of the city the predicted risk of having antibodies against SARS-CoV-2 varies between 10–15%. In the north-west and south-east areas, it was higher than 15%, while, areas with a lower risk (< 2.5%) were observed in the north-east zone. The conurbation of Coquimbo-La Serena (Fig. 4) showed a similar heterogeneity to Santiago. The highest risk (10–15%) was found in the northern part of La Serena and on a peninsula in southern Coquimbo.,. In contrast, lower risk (< 2.5%) corresponds to the central area of La Serena and the coast urbanized area. The city of Talca (Fig. 5) showed less heterogeneity in the model results and in distribution of analyzed ecological variables.

**Fig. 6:**
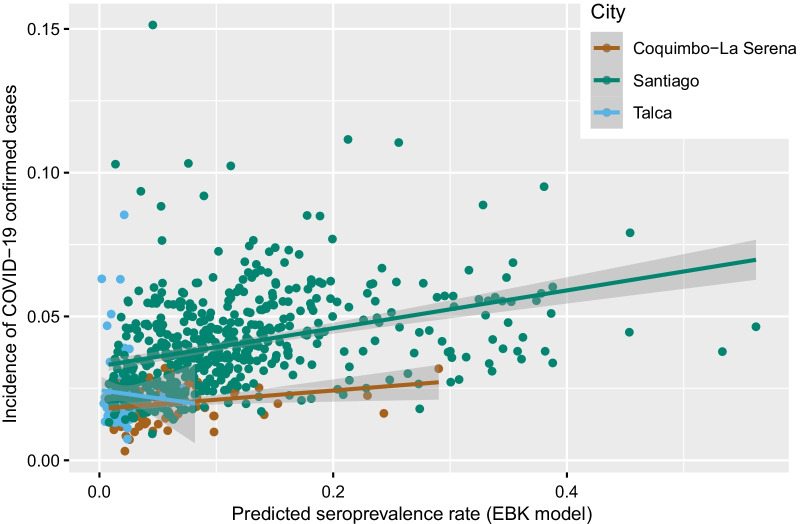
Correlation between incidence of COVID-19 confirmed cases and seroprevalence rate from Empirical Bayesian Kriging model. Chile 2020

The correlation analysis showed a significant association between the predicted risk of infection (EBK model) and the MOH reported cumulative incidence rate in Santiago (r = 0.449) and in Coquimbo-La Serena (r = 0.256). While in Talca there was no significant association (r = 0.06) (Fig. 6).

## Discussion

Our study shows the prevalence of SARS-CoV-2 antibodies in Chile at approximately 10% 267 days after the first imported case was detected, varying from 2 to 11% in three different cities, a figure nearly three times higher than the incidence rate reported by the Chilean Ministry of Health (MOH).

This heterogeneous prevalence inside Chile is consistent with other seroprevalence studies; for instance, Spain showed 5% (95% CI 4.7–5.4) with substantial geographic variation, ranging from more than 10% in Madrid to less than 3% in some coastal areas [30]. In Brazil, seroprevalence measured in June 2020 varies among cities from 0% to 25.4% [31].

Our study found similar antibody prevalence between sexes, slightly higher in males but not statistically significant. Comparable results between sexes were reported in several population-based studies [4, 11]. Also, we found no significant differences between age groups. Interestingly, children showed the same seroprevalence as other age groups despite national school closure ten days after the first case was detected [2]. A possible explanation for the high prevalence in children is that, with a relatively high level of population contagion, despite the suspension of school activities, minors were infected within homes, which is consistent with the high rate of attack within the homes.

On the other hand, the prevalence of older people is lower, showing some isolation from people of this age group. A similar phenomenon in children was also observed in Iquitos, Peru [20]. The reported OR for children infection aged < 18 in studies of household contacts was 0.77 (95% CI 0.27–2.17) in Italy and 1.39 (95% CI 0.55–3.53) in Utah, and Wisconsin in the USA [32, 33]. The infection rate in children is still debated around the globe.

In our study, a high proportion (63.9%) of people who developed antibodies reported symptoms. Nevertheless, a third remained asymptomatic, which was significantly higher in people below 15 years old. In Lima, in the first epidemic wave, 56.1% cases were asymptomatic and in Spain 32.7%, which may reflect the variation in expression of COVID-19 worldwide [22, 30].

Nearly 40% of symptomatic cases sought medical care, which may explain that many remained unreported, resulting in undetected ongoing transmission. This behavior could be related to health care access barriers, including cultural barriers associated with interpreting medical signs or symptoms, delaying consulting, and worsening disease outcomes.

Smoking showed a negative association with SARS-CoV-2 infection. This paradoxical effect has been associated with an underrepresentation of active smokers [34]. In that sense, our study detected that people with previous COVID-19 diagnoses reported significantly lower current tobacco consumption than those without the disease. However, the protective effect of tobacco use has also been evidenced in European countries, reporting a significant negative association between smoking prevalence and the prevalence of COVID-19 across the 38 European nations after controlling for confounding factors (p = 0.001) [35]. Some authors propose that nicotine prevents infection by competing with the virus with the ACE2 receptor. In this sense, epidemiological and experimental evidence has been found [36, 37].

In sum, we found that 10.4% of people were infected, almost 5% self-reported COVID-19 diagnosis (120/2493), and 0.4% (11/2493) were hospitalized; practically one in ten self-reported confirmed cases. As expected, the main risk factor was contact with confirmed cases. However, only 60% were quarantined, evidencing failures in contact tracing.

We couldn't find evidence of the association between seroprevalence and overcrowding at the individual level. However, we did find an association at the ecological level between seroprevalence and residential density (people/dwelling). Moreover, the sampling approach enabled us to contact more than one household member, thus facilitating the study of transmission within households. The secondary attack rate (SAR) was 38.4%. A systematic review (43 articles) estimated a pooled SAR of 18.1% (95% CI 15.7–20.6) ranging from 3.9% to 549% [38]. This high SAR is consistent with our finding of higher seroprevalence among people referring contact with confirmed cases at home, compared to contact in other places. Therefore, the critical role of household transmission needs to be considered when confinement measures are implemented, such as facilities to isolate cases away from the household.

The IFR in the three cities ranged from 0.59% to 2.58% for COVID-19 confirmed deaths, with an overall ratio of 1.3%. Talca exhibited a higher lethality related to reduced found seropositivity, giving an unreliable figure. Santiago doubled the Coquimbo-La Serena estimate (IFR 1.32 vs. 0.59), coincident with larger outbreak affecting the capital during 2020. In Santiago, we observed an abrupt rise in mortality and substantial stress on hospital capacity, unlike situations in the other two cities until 2021 [3]. Ioannidis estimated IFR based on seroprevalence data of 61 studies ranging from 0.0 to 1.63%, in the range of our pooled results [39].

Spatial analysis demonstrated the strong association between risk of infection and social determinant distribution, e.g., education and population density, in the territory, which was clearly observed in Santiago and Coquimbo-La Serena (larger and more socially segregated than Talca), with crowded and low-income neighborhoods where the virus transmits more easily [5]. In the case of Santiago, the interpolation shows that census zones with higher levels of socio-educational vulnerability show a seroprevalence six times higher than the wealthiest neighborhoods. Coquimbo-La Serena has four to six times more seroprevalence in sectors with greater socio-educational vulnerability than higher socio-economic levels.

We found a significant correlation between our spatial model of infection risk and the official registered cases by census zone in two of the three cities. Moreover, the residuals (zones with higher deviation) in Santiago pointed out areas that Mena suggested have less access to COVID-19 diagnostic tests [8]. So, the model was able to identify higher infection risk areas within two of the three cities.

The heterogeneity of infection risk distribution is consistent with the structural social inequalities that characterize Chile. Urban segregation is one of the most evident effects, a common phenomenon in larger cities throughout Latin America [40, 41]. Also, at the individual level, higher seroprevalence was associated with lower education and public health insurance (a proxy for middle and low-income levels in Chile). This social disparity was also reported in Lima, where the less advantageous tripled seroprevalence of the wealthier [22].

Thus, both at the individual level and in the spatial analysis, our results provide evidence of social determinants of COVID-19, and we note similarities with other respiratory transmitted diseases considering five levels of analysis [42]. First, social stratification, which affects the probability of contact with a contagious case. Second, the physical and social environment determining the exposure to the virus is inversely related to social position; overcrowding, job insecurity, dwelling conditions, and mobility are related to transmission. Third, vulnerability to infection and serious illness also have social and cultural modulation related to distribution of comorbidities. Fourth, unequal access to diagnostic testing and, late, to therapeutic resources, affect transmission and serious infection. The fifth level of analysis is the difference in consequences, not only of fatalities but also of social impact of deaths, catastrophic expending, loss of future income due to persistent sequelae, which we have not explored in our study.

Our primary limitation was the low number of cases in Talca, perhaps due to the lack of statistical power or the use of a higher proportion of rapid tests (9.9%) with a lower sensibility. Other limitations of our study are potential bias: first, recall bias of self-reported symptoms and diagnosis; second, the natural decrease of antibodies after infection may have biased the results toward lower prevalence and may explain the finding of almost 20% of seronegative results among those who self-reported COVID-19. And third, survivor bias, as we inquired about deaths of household members due to COVID-19, finding two deaths, both males over 60 years old. Finally, as in any model, EBK has limitations; thus, to validate, we compared distribution of our seroprevalence results with incidence of COVID-19 confirmed cases by census zone reported to MOH, finding a significant correlation.

To our knowledge, this is the first population-based SARS-CoV-2 seroprevalence study carried out in three cities of Chile. Strengths of this study include the high response rate and the interdisciplinary approach incorporating geographic modeling. Our results enabled us to estimate the real burden of SARS-CoV-2 infection in three cities and identify the spatial distribution of disease risk and areas with relative lack of access to diagnostic tests. All this data and results can help guide the design of strategies and prioritize intervention areas in urban contexts, not only for SARS-CoV-2 but for other infectious diseases as well.

## Conclusions

Our study found the proportion of people with symptomatic infection, those seeking medical care, hospitalizations among confirmed cases, and the infection fatality ratio. Additionally, we evaluated quarantine coverage and as primary and secondary attack rate in households. As expected, antibody presence was higher in people with previous diagnoses of COVID-19, in people in contact with confirmed cases, and in those with COVID-19 compatible symptoms. The most significant infection risk was household contact with confirmed cases. Seroprevalence was significantly associated with lower level of education, public health insurance, and population density.

Our results reveal highly heterogeneous spread of COVID-19 in urban contexts and significant association between seroprevalence and social determinants. The spatial model enabled us to identify high transmission risk areas in different Chilean urban centers, those not detected by surveillance and health care systems and relatively neglected.

In sum, at the end of 2020, besides being very far from any herd immunity level, the seroprevalence shows that the first wave in Chile was remarkable. This is consistent with the severe stress observed throughout the health care system and the excess of mortality, evidencing the impact of COVID-19 on naive populations. In such situations, government authorities must promote interdisciplinary analysis and intersectoral interventions to reduce vulnerability for future waves, especially in highly populated urban contexts.

## Supplementary Information

**Additional file 1.** **Table S1.** Sample and population distribution in the three cities. **Table S2.** Population seroprevalence of SARS-CoV-2 antibodies by city and sociodemographic variables. Chile 2020. **Table S3.** Seroprevalence of SARS-CoV-2 antibodies according to contact history and clinical characteristics by city. Chile 2020. **Table S4.** Frequency and type of symptoms and presence of SARS-CoV-2 antibodies by city. Chile 2020.** Table S5.** Seroprevalence of SARS-CoV-2 antibodies according to self-reported chronic conditions. Chile 2020. **Table S6.** Seroprevalence of SARS-CoV-2 antibodies according to self-reported chronic conditions by city. Chile 2020.

## Data Availability

Data collected for the study, including individual participant data (deidentified) and data dictionary defining each field in the set, are available from the corresponding author on reasonable request.
