# Supplementary material for: Seroprevalence, spatial distribution, and social determinants of SARS-CoV-2 in three urban centers of Chile
Source: BMC Infect Dis. 2022 Jan 28;22:99. doi: 10.1186/s12879-022-07045-7 (PMC8795965; doi:10.1186/s12879-022-07045-7)
Supplement: Supplementary file 1 — Additional file 1. Table S1. Sample and population distribution in the three cities. Table S2. Population seroprevalence of SARS-CoV-2 antibodies by city and sociodemographic variables. Chile 2020. Table S3. Seroprevalence of SARS-CoV-2 antibodies according to contact history and clinical characteristics by city. Chile 2020. Table S4. Frequency and type of symptoms and presence of SARS-CoV-2 antibodies by city. Chile 2020. Table S5. Seroprevalence of SARS-CoV-2 antibodies according to self-reported chronic conditions. Chile 2020. Table S6. Seroprevalence of SARS-CoV-2 antibodies according to self-reported chronic conditions by city. Chile 2020. [file 12879_2022_7045_MOESM1_ESM.docx]

**Supplementary Information**

**Sample estimation for Santiago (Metropolitan Region. Chile).**

**Considering 34 urban municipalities. School of Government of the Universidad del Desarrollo surveys**

Assuming unknown population. the sample size was estimated with maximum variance and simple random sampling assuming that we do not know the size of the population. First. we used the maximum variance of the margin of error of an interval with 95% confidence.

| $n=\frac{z_{\frac{\alpha}{2}}^{2}*p*(1-p)}{{ME}^{2}}$ = 384. where | $z_{\frac{\alpha}{2}}^{2}:$ Confidence level of the interval.  $p:$ Expected proportion of the quality to be evaluated.  $ME:$Margin of error of the confidence interval |
| --- | --- |

The standard error of an estimate from a complex sample design will be larger than the standard error of a simple random sample of the same size. N. The proportional increase in the standard error of an estimate due to the use of a complex design or design effect (DEFF) can be defined as:

| $DEFF=\frac{EE(complex sampling of size N)}{EE (SRS sampling of size N)}$ | $n=\frac{z_{\frac{\alpha}{2}}^{2}*p*\left( 1-p \right)*{DEFF}^{2}}{{ME}^{2}}$ |
| --- | --- |

Therefore. a complex sample can be expressed as:

| **Estimated proportion (p)** | **Dessign effect** | **Confidence level (95%)** | **Error** | **Sample (n)** |
| --- | --- | --- | --- | --- |
| 0.5 | 1.0 | 1.96 | 0.030 | 1.067 |
| 0.5 | 1.4 | 1.96 | 0.040 | 1.176 |
| 0.5 | 1.8 | 1.96 | 0.050 | 1.176 |

**Systematic sampling for housing selection in a block**

The process of selection of dwellings in each randomly selected block is was organized as follows.

1. Sampling frame: Preparation of a list of blocks and dwellings based on the 2012 Census.
2. The dwellings in each block correspond to a sample frame (for that block).
3. Division of the sampling frame into n groups of dwellings. The size of these groups will be: K=N/n. where K is called the interval or lift coefficient.
4. Start number: we obtain a random integer A. less or equal to the interval. This number will correspond to the first dwelling that we will select for the sample within the first group of dwellings in which we have divided the sampling frame.
5. Selection of the remaining n-1 dwellings: We select the following dwellings from the randomly selected dwelling. by means of an arithmetic succession. selecting the dwellings from the rest of the groups into which we have divided the sample that occupy the same position as the initial dwelling. This is equivalent to saying that we will select the dwellings:

**A; A + K; A + 2K; A + 3K; ....; A + (n-1)K**

1. As a replacement. in the case of an unoccupied dwelling or lack of interest in participating. we consider an interval of +/- 1 dwelling. so that its direct neighbor can replace it.

**Selection of individuals within the dwelling**

A table was included in the questionnaire to randomly select a person in the dwelling. Its use is described in Survey Sampling (Kish. 1995). and is known as a "Kish Table". The Kish method (Mkish) provides a random sampling method to select one person from each dwelling. regardless of the sampling method used to select them.

**Sample weights calculation**

**SARS-CoV-2 antibodies prevalence survey in Santiago (Metropolitan Region). La Serena-Coquimbo (Coquimbo Region) and Talca (Maule Region) Chile 2020.**

The survey was carried out in the urban area of the Metropolitan Region (Santiago) and the conurbations defined by the municipalities of La Serena-Coquimbo and Talca-Maule. The sampling frame that gave rise to the sample selection is available and the composition of the population under study is obtained from the Census 2017. and the official population projections of the National Statistics Institute (INE) for the year 2020. which corresponds to the reference year of the ANID survey.

The sampling frame corresponds in all three cases to a frame of areas. defined by the blocks of the urban area. updated with information from the 2016 PreCensus. In this context. the design weights considers the type of sample implemented. which differs for each study domain. The process of calculating the design weight for each domain is summarized below.

Santiago

In the case of the Santiago City. this is a two-phase sampling. The first phase corresponds to a probability sampling. stratified in three selection stages. with the following characteristics:

Target population 1st. phase: people 18 years of age or older in the Province of Santiago and the Municipalities of Puente Alto and San Bernardo.

Stratification 1st phase: the strata are made up of the 34 urban municipalities belonging to the study domain.

Sampling stages 1st phase:

- First stage: blocks that make up the sampling frame provided by INE.

- Second stage: Dwellings belonging to the selected block.

- Third stage: Person 18 years of age or older belonging to the dwellings selected in the previous stage.

The second phase corresponds to a probability sampling under simple random sampling of individuals who have been selected in the first sampling phase. For this study domain. the sample of dwellings of the survey is composed of the dwellings corresponding to the individuals selected in this second selection phase. On the other hand. the sample of persons is made up of all individuals 7 years of age or older present in these dwellings.

**Design weights calculation**

As mentioned above. the construction of the design weights considers the sample design that gave rise to the sample. therefore. the following steps are followed:

1. Determine the first-phase selection probability. which corresponds to the conditional selection probability of the individual aged 18 years or older. given that a dwelling has been selected within a specific block of a commune belonging to the study domain.
2. Determine the second-phase selection probability. which corresponds to the conditional selection probability of the dwelling for the survey. given that it was selected in the first phase.
3. Determine the probability of selection of all members aged 7 years or older in the dwelling selected in the second phase. for which it is assumed that all have the same probability of selection calculated in the second phase.
4. The design weights adjusted for non-response is obtained for each individual in the household as the inverse of the probability determined in the previous step. corrected for members 7 years of age or older who respond.
5. Finally. the design weights are calibrated based on the population projections for the 2020 reference year. The complete post-stratification method is used to cross the variables gender and two age brackets (15-39 and 40+).

**La Serena-Coquimbo and Talca-Maule Conurbations**

In the case of the conurbations. this is a probability sampling. stratified in two selection stages. whose characteristics are as follows:

Target population: people aged 7 years or older in the conurbations of La Serena-Coquimbo and Talca-Maule.

Stratification: the strata are made up of the census districts of the municipalities belonging to the study domain.

Sampling stages:

- First stage: blocks that make up the sampling frame provided by INE.

- Second stage: Dwellings belonging to the selected block.

The sample of individuals for the ANID survey of these study domains is composed of all persons 7 years of age or older in the selected dwellings.

**Design weights calculation**

The following steps are followed to construct the design weights:

1. Determine the conditional selection probability of the dwellings belonging to each selected block within a specific census stratum.
2. Determine the probability of selection of all members 7 years of age or older in the selected dwelling. for which it is assumed that all have the same probability of selection calculated for the dwelling.
3. The adjusted for non-response design weights are obtained for each individual in the household as the inverse of the probability determined in the previous step. corrected for members 7 years of age or older who respond.
4. Finally. the design weights are calibrated based on the population projections for the 2020 reference year. In this case. they are calibrated by the complete post-stratification method for the crossing of the commune and gender variables.

**Table S1. Sample and population distribution in the three cities.**

|  | | **Santiago** | **Talca** | **Coquimbo - La Serena** | **Total** |
| --- | --- | --- | --- | --- | --- |
| Percentage of women | Sample | 59,2% | 59,4% | 64,6% | **60,3%** |
|  | Population (CENSUS 2017) | 50,8% | 51,8% | 51,9% | **50,9%** |
| Mean age | Sample | 40,5 | 43,5 | 42,7 | **41,6** |
|  | Population (CENSUS 2017) | 39,5 | 42,7 | 44,1 | **39,9** |
| Proportion of individuals with university education | Sample | 26.0% | 16.2% | 15.1% | **21.6%** |
|  | Population (CENSUS 2017) | 20.4% | 16.7% | 15.5% | **20.0%** |
| Native South American ethnicity | Sample | 9.6% | 7.1% | 10.7% | **9.2%** |
|  | Population (CENSUS 2017) | 10.3% | 4.3% | 8.6% | **10.0%** |

**The spatial interpolation method (Empirical Bayesian Kriging)**

We estimated the probabilities of SARS-CoV-2 infection at the individual level using the multivariate model. The infection probability was geolocated according to the address of each sampled household (anonymized) (Table 7 in the manuscript).

Due to the spatial nature of the data and considering that they do not have representativeness at the municipal scale. an interpolation of the individual model results was carried out for each of the cities. using a method called Empirical Bayesian Kriging (EBK) in ArcGis 10.7 software.

The EBK is a geostatistical technique that has the advantage of automatically estimating various adjustment parameters (such as area of ​​influence or number of neighbors to analyze) from the data set used through the creation of multiple subsets and simulations that facilitate prediction (ESRI. 2020).

The EBK is useful for working with spatial databases that do not have a clear distribution pattern. levels of spatial asymmetry. or do not present spatial autocorrelation (Webster. Oliver. 2007). It allows making predictions of non-stationary data. with lower standard errors. even working with smaller datasets. Based on the above. and considering the complexity of the sample obtained. we choose this tool to interpolate the results of the multivariate model for the three cities analyzed. using the following parameters for each town:

**Parameters for Empirical Bayesian Kriging models**

| **Variable** |  | **Study area** | |
| --- | --- | --- | --- |
|  | **Coquimbo - La Serena** | **Santiago** | **Talca** |
| Methodology | EBK | EBK | EBK |
| Total sampling points | 478 | 1.441 | 572 |
| Minimum value | 0.006 | 0.008 | 0.002 |
| Maximum value | 0.497 | 0.756 | 0.265 |
| Used setting | Standard Circular  4 sections in 45° | Standard Circular  4 sections in 45° | Standard Circular  4 sections in 45° |
| Sampling distance | 600 meters | 600 meters | 300 meters |
| Number of neighbors  (min – max) | 10 - 15 | 10 - 15 | 10 - 15 |
| Semivariogram type | Power | Power | Power |
| Total of simulations | 100 | 100 | 100 |

The resulting models represent the individual risk predicted by the EBK model (weighted means) at the 2017 census area scale. The models were published in a territorial information platform with free access for all public. available at <https://arcg.is/1nT5CT0>

The following information is available on the website for the three study areas: 1) the sample distribution (anonymized); 2) the main census variables used to formulate the multivariate model (educational level. residential density. and overcrowding). and 3) the spatialization of the model developed using empirical Bayesian kriging for the three cities.

References:

- Esri (2020). ArcGIS (Version 10.5). Esri Inc. <https://www.esri.com/en-us/arcgis/products/arcgis-desktop/>
- Webster. R.. Oliver. M.A.. 2007. Geostatistics for Environmental Scientists. Wiley. Chichester.

**Table S2. Population seroprevalence of SARS-CoV-2 antibodies by city and sociodemographic variables. Chile 2020**

|  | | La Serena - Coquimbo | | | Talca | | | Santiago | | |
| --- | --- | --- | --- | --- | --- | --- | --- | --- | --- | --- |
|  |  | Seropositives/ Participants (n) | Weighted seroprevalence % (95%CI) * | OR (95%CI) * | Sample seropositives (n) | Weighted seroprevalence % (95%CI) * | OR (95%CI) * | Sample seropositives (n) | Weighted seroprevalence % (95%CI) * | OR (95%CI) * |
|  | Overall | 30/478 | 5.6 (3.3-9.5) |  | 12/574 | 2 (0.8-4.7) |  | 200/1441 | 11 (8.2-14.7) |  |
| Sex | Male | 11/169 | 5.6 (2.9 -10.4) | 0.99 (0.5-2.0) | 5/233 | 2.6 (0.9-7.8) | 1.9 (0.6-5.6) | 91/588 | 13 (8.7-18.8) | 1.5 (0.9-2.5) |
|  | Female | 19/309 | 5.6 (3.0-10.2) | ref. | 7/341 | 1.4 (0.6-3.3) | ref. | 109/853 | 9.1 (6.3-12.9) | ref. |
| Age | 7 – 14 | 3/36 | 4.6 (1.1-17.7) | ref. | 0/51 | 0 (NA) | NA | 18/89 | 14.1 (6.7-27.1) | ref. |
|  | 15 – 24 | 5/96 | 4.0 (1.6 -9.7) | 0.9 (0.2-4.2) | 2/75 | 2.1 (0.4- 9.1) | NA | 31/189 | 9.9 (5.5-17.2) | 0.7 (0.2-1.9) |
|  | 25 – 39 | 8/88 | 6.1 (2.5-13.9) | 1.4 (0.3-5.5) | 2/116 | 0.8 (0.2-3.3) | NA | 52/445 | 10.8 (6.7-17.0) | 0.7 (0.3-1.9) |
|  | 40 – 59 | 11/136 | 11.3 (5.6-21.3) | 2.6 (0.5-14.1) | 6/183 | 4.1 (1.4-11.7) | NA | 76/494 | 12.5 (7.8-19.3) | 0.9 (0.4-2.1) |
|  | ≥ 60 | 3/122 | 1.8 (0.4-7.5) | 0.4 (0.1-3.1) | 2/149 | 1.2 (0.3-4.9) | NA | 23/224 | 7.9 (3.8-15.7) | 0.5 (0.2-1.6) |
| Nationality | Chilean | 26/464 | 5.4 (3.0-9.3) | ref. | 10/556 | 1.8 (0.7-4.7) | ref. | 185/1358 | 9.9 (7.4-13.1) | ref. |
|  | Others | 4/14 | 13 (1.7-55.6) | 2.6 (0.3-23.9) | 2/18 | 8.7 (2.6-25.2) | 5.2 (1.1-25.5) | 15/83 | 21.9 (9.0-44.1) | 2.6 (0.9-7.4) |
| Native South American Ethnicity (by self-identification) | Yes | 0/50 |  | NA | 0/41 | 0 (0) | NA | 23/139 | 19.6 (7.7-41.8) | 2.1 (0.7-6.5) |
|  | No | 30/428 | 6.1 (3.6-10.3) | NA | 12/533 | 2.1 (0.9-5.1) | NA | 177/1302 | 10.3 (7.6-13.9) | ref. |
| Educational level (for participants > 17 years) (n=2.233) | None or primary | 1/59 | 1.0 (0.1-7.1) | 0.2 (0.02-1.7) | 1/85 | 0.9 (0.1-6.2) | 0.8 (0.1-9.6) | 9/90 | 5.2 (1.8-14.3) | 0.8 (0.2-2.7) |
|  | Secondary | 18/227 | 7.4 (3.8-14.0) | 1.2 (0.3-4.5) | 8/283 | 3.1 (1.2-7.8) | 2.8 (0.5-14.4) | 104/620 | 15.3 (10.6-21.6) | 2.4 (1.2-5.0) |
|  | Technical | 4/59 | 5.6 (1.6-17.8) | 0.9 (0.2-5.4) | 1/44 | 2.0 (0.3-14.0) | 1.8 (0.1-22.7) | 33/227 | 10.0 (5.3-18.3) | 1.5 (0.6-3.8) |
|  | University | 4/73 | 6.2 (1.9-18.7) | ref. | 2/93 | 1.1 (0.3-4.7) | ref. | 30/374 | 6.9 (3.7-12.3) | ref. |
| Health Insurance (n=2.347) | Public | 29/384 | 6.4 (3.6-11.0) | 2.1 (0.3-14.9) | 9/476 | 1.9 (0.7-5.3) | 0.6 (0.1-6.2) | 154/951 | 13.8 (9.9-18.8) | 2.7 (1.3-5.6) |
|  | Private | 1/63 | 3.1 (0.5-16.9) | ref. | 2/53 | 3.2 (0.4-20.2) | ref. | 41/420 | 5.6 (2.9-10.2) | ref. |
| Overcrowding (n=2.487) | No | 29/453 | 5.5 (3.2-9.4) | ref. | 9/544 | 1.8 (0.6-4.7) | ref. | 181/1341 | 11.4 (8.3-15.3) | ref. |
|  | Yes | 1/25 | 6.9 (0.9-36.9) | 1.3 (0.2-10.7) | 3/30 | 5.1 (0.7-29.5) | 3.0 (0.3-29.9) | 19/94 | 7.6 (2.7-19.6) | 0.7 (0.2-2.0) |
| Number of residents | <5 | 24/318 | 6 (3.2-10.9) | ref. | 6/386 | 1.7 (0.6-4.9) | ref. | 103/947 | 9.8 (6.4-14.7) | ref. |
|  | ≥5 | 6/160 | 4.9 (1.7-13.3) | 0.8 (0.2-2.8) | 6/188 | 2.6 (0.6-10.6) | 1.6 (0.2-9.8) | 97/494 | 13.9 (9.5-20.0) | 1.5 (0.8-2.8) |
| Type of dwelling (n=2.486) | House | 29/470 | 5.6 (3.3-9.6) | NA | 12/565 | 2.1 (0.9-5.0) | NA | 160/1107 | 11.4 (8.3-15.5) | ref. |
|  | Department | 0/5 | NA | NA | 0/9 | NA | NA | 38/330 | 10.6 (6.1-17.7) | 0.9 (0.5-1.8) |
| Heating fuel (n=2.486) |  |  |  |  |  |  |  |  |  |  |
| Gas | No | 27/326 | 7.4 (4.2-12.7) | ref. | 9/363 | 2.4 (0.9-6.6) | ref. | 84/626 | 10 (6.8-14.4) | ref. |
|  | Yes | 3/152 | 1.5 (0.3-6.9) | 0.2 (0.03-1.0) | 3/211 | 1 (0.3-3.3) | 0.4 (0.1-2.0) | 116/815 | 12 (7.7-18.2) | 1.2 (0.7-2.3) |
| Kerosene | No | 30/460 | 5.8 (3.4-9.7) | NA | 8/433 | 1.7 (0.5-5.2) | ref. | 143/1014 | 11.2 (7.8-15.8) | ref. |
|  | Yes | 0/18 | NA | NA | 4/141 | 2.7 (0.7-10.3) | 1.6 (0.3-10.0) | 57/427 | 10.6 (6.2-17.5) | 0.9 (0.5-1.9) |
| Fire Wood | No | 22/423 | 4.2 (2.1-8.2) | ref. | 8/315 | 2.4 (0.9-6.1) | ref. | 199/1437 | 11 (8.2-14.7) | ref. |
|  | Yes | 8/55 | 14.3 (6.0-30.4) | 3.8 (1.2-12.4) | 4/259 | 1.5 (0.3-8.9) | 0.6 (0.1-5.1) | 1/4 | 23.9 (2.6- 78.7) | 2.5 (0.2-30.5) |
| Electricity | No | 16/337 | 4.5 (2.2-8.7) | ref. | 8/377 | 2.3 (0.7-7.0) | ref. | 135/933 | 12.4 (8.5-17.9) | ref. |
|  | Yes | 14/141 | 8.4 (3.6-18.4) | 2 (0.6-6.1) | 4/197 | 1.6 (0.5-5.3) | 0.7 (0.1-3.8) | 65/508 | 9 (5.7-13.9) | 0.7 (0.4-1.3) |

*Weighted for sampling weights. OR=odds ratio. (ref)=reference category. 95% CI = 95% confidence interval.

**Table S3. Seroprevalence of SARS-CoV-2 antibodies according to contact history and clinical characteristics by city. Chile 2020**

|  | | La Serena - Coquimbo | | | Talca | | | Santiago | | |
| --- | --- | --- | --- | --- | --- | --- | --- | --- | --- | --- |
|  |  | Seropositives/ Participants (n=478) | Weighted seroprevalence % (95%CI) * | OR (95%CI) * | Seropositives/ Participants (n=574) | Weighted seroprevalence % (95%CI) * | OR (95%CI) * | Seropositives/ Participants (n=1.141) | Weighted seroprevalence % (95%CI) * | OR (95%CI) * |
| COVID-19 diagnosis (n=2.493) | No | 14/455 | 2.5 (1.2-5.1) | ref. | 8/569 | 1.5 (0.6-3.8) | ref. | 122/1349 | 6.9 (5.0-9.5) | ref. |
|  | Yes | 16/23 | 57.2 (28.2-81.9) | 52.1 (13.1-207.8) | 4/5 | 95.8 (68.5-99.6) | 1488.3 (138.1-16037.0) | 78/92 | 69.4 (42.3-87.5) | 30.5 (9.4-99.2) |
| COVID-19 hospitalization (n=120) | No | 14/21 | 52.9 (13.6-88.9) | NA | 4/5 | NA | NA | 70/83 | 70.4 (50.3-84.8) | ref. |
|  | Yes | 2/2 | NA | NA | 0/0 | NA | NA | 8/9 | 56.4 (38.9-72.5) | 0.5 (1.2-1.7) |
| Contact with confirmed cases (n=2.492) | No | 12/434 | 3 (1.4-6.3) | ref. | 3/542 | 0.8 (0.2-3.0) | ref. | 120/1221 | 8 (5.6-11.1) | ref. |
|  | Yes (≥ 1 person) | 18/44 | 29.9 (15.4-50.1) | 24.3 (10.6-55.8) | 9/32 | 28.3 (10.4-57.3) | 70.3 (17.8-277.1) | 80/220 | 31.3 (19.7-45.8) | 5.2 (3.8-7.3) |
| Risk contact place (n=296) | At home | 12/34 | 25.3 (10.5-49.4) | 0.5 (0.1-2.7) | 6/11 | 64.8 (22.9-91.9) | 17.1 (1.4-212.3) | 64/152 | 42.8 (27.0-60.3) | 4.3 (1.1-16.2) |
|  | Work /small gatherings/others | 6/10 | 38.2 (18.8-62.2) | ref. | 3/21 | 9.7 (3.0-27.5) | ref. | 16/68 | 15 (5.4-35.3) | ref. |
| Quarantine (n=296) | No | 1/16 | 8.2 (6.4-10.3) | ref. | 2/15 | 6.9 (1.5-26.1) | ref. | 29/92 | 22.7 (11.0-41.0) | ref. |
|  | Yes | 17/28 | 51.9 (27.5-75.4) | 12.1 (3.4-42.8) | 7/17 | 42.6 (14.4-76.6) | 10 (0.7-138.5) | 51/128 | 40.4 (23.5-60.0) | 2.3 (0.7-7.2) |
| Any symptom compatible with COVID-19† | No | 12/412 | 2.7 (1.3-5.2) | ref. | 6/468 | 1.2 (0.4-3.6) | ref. | 71/961 | 5.6 (3.7-8.5) | ref. |
|  | Yes | 18/66 | 26 (13.7-43.8) | 12.7 (5.0-32.2) | 6/106 | 5.5 (1.8 -15.8) | 4.6 (1.2-17.7) | 129/480 | 23.9 (17.1-32.4) | 5.3 (3.1-9.1) |
| Number of symptoms compatible with COVID-19 | None | 12/412 | 2.7 (1.3-5.2) | ref. | 6/468 | 1.2 (0.4-3.6) | ref. | 71/961 | 5.6 (3.7-8.5) | ref. |
|  | 1-2 symptoms | 2/26 | 4.8 (1.0-20.5) | 1.8 (0.3-10.2) | 3/66 | 4.2 (1.2-14.2) | 3.5 (0.6-19.5) | 27/183 | 20.1 (10.2-35.7) | 4.2 (1.7-10.1) |
|  | 2 -3 symptoms | 4/14 | 19.4 (6.0-47.8) | 8.7 (2.3-32.6) | 1/21 | 5.9 (0.8-32.8) | 4.9 (0.7-32.9) | 41/160 | 19.7 (11.5-31.8) | 4.1 (1.9-8.8) |
|  | ≥ 5 symptoms | 12/26 | 52.1 (28.3-75.1) | 39.3 (12.4-124.7) | 2/19 | 10.5 (2.0-40.3) | 9.3 (1.7-49.8) | 61/137 | 37.9 (23.7-54.6) | 10.3 (4.9-21.4) |
| Seek medical care (n=651) | No | 8/40 | 15.5 (5.1-38.6) | ref. | 1/70 | 1.0 (0.1-7.6) | ref. | 55/289 | 18.2 (11.7-27.1) | ref. |
|  | Yes | 10/25 | 41.2 (18.1 -69.0) | 3.8 (0.7-18.9) | 5/36 | 15.5 (4.5-41.4) | 17.6 (1.4-219.7) | 74/191 | 32.2 (21.1-45.8) | 2.1 (1.1 -4.3) |
| Tobacco consumption | No | 25/353 | 7.1 (4.6-10.3) | ref. | 8/408 | 2.0 (0.9-3.8) | ref. | 154/1003 | 15.4 (13.1-17.7) | ref. |
|  | Yes | 5/125 | 4 (1.3-9.1) | 0.5 (0.2-1.5) | 4/166 | 2.4 (0.6-6.1) | 1.2 (0.4-4.2) | 44/435 | 10.1 (7.4-13.3) | 0.6 (0.4-0.9) |

*Weighted for sampling weights. OR=odds ratio. (ref)=reference category. 95%CI = 95% confidence interval. † Including symptoms of fever. cough. odynophagia. dyspnea. headache. myalgia. chest pain. abdominal pain. diarrhea. fatigue. anosmia and dysgeusia.

**Table S4. Frequency and type of symptoms and presence of SARS-CoV-2 antibodies by city. Chile 2020.**

|  | La Serena - Coquimbo | | | | Talca | | | | Santiago | | | |
| --- | --- | --- | --- | --- | --- | --- | --- | --- | --- | --- | --- | --- |
|  | Sample (n=478) | Symptoms in seropositive (%)* | Symptoms in seronegative (%)* | OR  (95%CI)† | Sample (n=574) | Symptoms in seropositive (%)* | Symptoms in seronegative(%)* | OR  (95%CI)† | Sample (n=1.441) | Symptoms in seropositive (%)* | Symptoms in seronegative (%)* | OR  (95%CI)† |
| Fever | 21 | 34.6 | 3.0 | 17.1 (5.7-51.4) | 21 | 20.2 | 2.7 | 9.3 (1.4-63.7) | 131 | 24.6 | 5.0 | 6.2 (3.1-12.4) |
| Cough | 25 | 36.3 | 2.6 | 21.1 (8.3- 55.9) | 35 | 15 | 5.1 | 3.3 (0.9-12.4) | 177 | 26.8 | 8.7 | 3.8 (1.7-8.4) |
| Odynophagia | 35 | 23.7 | 6.8 | 4.2 (1.5-11.6) | 47 | 18.1 | 5.4 | 3.9 (1.0-15.3) | 244 | 29.8 | 12.8 | 2.9 (1.6-5.2) |
| Dyspnoea | 19 | 21.8 | 2.2 | 12.3 (4.2-36.4) | 25 | 20.2 | 3.5 | 6.9 (1.0-46.9) | 103 | 14.7 | 4.8 | 3.4 (1.6-7.2) |
| Headache | 51 | 48.1 | 6.1 | 14.2 (5.9-34.1) | 47 | 3.2 | 10.4 | 0.3 (0.04-2.1) | 336 | 46.2 | 16.9 | 4.2 (2.2-7.9) |
| Myalgia | 35 | 40.4 | 4.3 | 15.3 (6.1-38.1) | 38 | 15 | 5.9 | 2.8 (0.7-10.9) | 233 | 39.6 | 11.4 | 5.1 (2.9-9.2) |
| Chest pain | 11 | 16.8 | 0.8 | 24.5 (7.2-83.0) | 12 | 15 | 1.6 | 11.3 (2.7-47.1) | 85 | 22.5 | 3.4 | 8.3 (3.1-22.3) |
| Abdominal pain / diarrhea | 17 | 29.8 | 1.6 | 25.6 (7.0-93.9) | 21 | 23.3 | 2.3 | 12.7 (2.4-67.4) | 130 | 18.8 | 8.1 | 2.6 (1.2-6.0) |
| Fatigue / prostration | 20 | 28.1 | 2.4 | 16.0 (4.8-53.7) | 20 | 6 | 4.8 | 1.3 (0.1-14.7) | 141 | 24.9 | 5.7 | 5.5 (2.8-10.7) |
| Anosmia | 15 | 32.2 | 0.8 | 59.0 (13.9-250.3) | 8 | 16.3 | 1.0 | 19.3 (1.7-214.7) | 77 | 23.1 | 1.0 | 29.2 (10.9-77.9) |
| Dysgeusia | 13 | 34.7 | 0.2 | 331.5 (37.5-2929.7) | 10 | 24.7 | 1.1 | 30.1 (5.3-171.4) | 66 | 21.1 | 0.9 | 30.6 (10.2-92.3) |

*Weighted for sampling weights. OR=odds ratio. † Reference category=seronegative. 95%CI = 95% confidence interval.

**Table S5. Seroprevalence of SARS-CoV-2 antibodies according to self-reported chronic conditions. Chile 2020**

|  | | Participants  (n) | Sample seropositives (n) | Weighted seroprevalence % (95%CI) * | OR  (95%CI) |
| --- | --- | --- | --- | --- | --- |
|  |  |  |  |  |  |
| BMI (n = 2.174) | Underweight | 55 | 2 | 29.2 (5.5-75.8) | 4.4 (0.6-34.1) |
|  | Normal | 669 | 55 | 8.9 (5.3-14.7) | ref. |
|  | Overweight | 791 | 85 | 10.6 (7.2-15.3) | 1.2 (0.6-2.3) |
|  | Obesity | 659 | 78 | 12.1 (7.9-18.0) | 1.4 (0.7-2.9) |
| Any comorbidity | No | 1.422 | 144 | 10.3 (7.3-14.3) | ref. |
|  | Yes | 1.071 | 98 | 10.5 (6.9-15.8) | 1.0 (0.6-1.8) |
| Diabetes | No | 2.253 | 217 | 10.7 (7.9-14.2) | ref. |
|  | Yes | 240 | 25 | 7.6 (3.9-14.2) | 0.7 (0.3-1.5) |
| Cancer (n=2.492) | No | 2.409 | 235 | 10.4 (7.8-13.8) | ref. |
|  | Yes | 83 | 7 | 9.3 (2.8-26.7) | 0.9 (0.2-3.2) |
| Hypertension (n=2.491) | No | 1.981 | 204 | 10.6 (7.9-14.1) | ref. |
|  | Yes | 510 | 38 | 9.4 (5.3-16.2) | 0.9 (0.5-1.6) |
| Heart disease | No | 2.405 | 237 | 10.6 (7.8-13.8) | ref. |
|  | Yes | 88 | 5 | 9.4 (1.8-20.4) | 0.6 (0.2-2.3) |
| HIV/AIDS | No | 2.481 | 241 | 10.3 (7.8-13.5) | ref. |
|  | Yes | 12 | 1 | 2.6 (3.4-77.5) | 3.1 (0.3-28.7) |
| Asthma/COPD | No | 2.320 | 225 | 10.3 (7.6-13.7) | ref |
|  | Yes | 173 | 17 | 12.8 (6.0-25.1) | 1.3 (0.5-3.0) |
| Chronic kidney disease (n=2.492) | No | 2.462 | 238 | 10.4 (7.8-13.8) | ref. |
|  | Yes | 30 | 4 | 9.1 (2.6-27.4) | 0.9 (0.2-3.3) |
| Endocrine system disease | No | 2.217 | 222 | 10.8 (8.1-14.2) | ref. |
|  | Yes | 276 | 20 | 6.2 (26.8-13.7) | 0.6 (0.2-1.3) |
| Osteo-articular diseases | No | 2.416 | 234 | 10.4 (7.8-13.8) | ref. |
|  | Yes | 77 | 8 | 9.9 (3.8-23.8) | 0.95 (0.3-2.8) |
| Tobacco consumption (n=2.490) | No | 1.764 | 187 | 11.9 (8.8-16.0) | ref. |
|  | Yes | 726 | 53 | 6.6 (4.0-10.8) | 0.5 (0.3-0.9) |

*Weighted for sampling weights. OR=odds ratio. (ref)=reference category. 95%CI = 95% confidence interval.

**Table S6. Seroprevalence of SARS-CoV-2 antibodies according to self-reported chronic conditions by city. Chile 2020**

|  | | La Serena - Coquimbo | |  | Talca |  |  | Santiago |  |  |
| --- | --- | --- | --- | --- | --- | --- | --- | --- | --- | --- |
|  |  | Seropositives/ Participants (n=478) | Weighted seroprevalence % (95%CI) * | OR (95%CI) * | Seropositives/ Participants (n=574) | Weighted seroprevalence % (95%CI) * | OR (95%CI) * | Seropositives/ Participants (n=1.141) | Weighted seroprevalence % (95%CI) * | OR (95%CI) * |
| BMI (n=2.174) | Underweight | 0/10 | 0 | NA | 0/11 | 0 | NA | 2/34 | 5.9 (0.7-19.6) | 0.5 (0.1-2.2) |
|  | Normal | 4/126 | 3.2 (0.9-7.9) | ref. | 4/118 | 3.4 (0.9-8.5) | ref. | 47/425 | 11.1 (8.2-14.4) | ref. |
|  | Overweight | 11/141 | 7.8 (4.0-13.5) | 2.6 (0.8-8.3) | 7/186 | 3.8 (1.5-7.6) | 1.1 (0.3-3.9) | 67/464 | 14.4 (11.4-18.0) | 1.4 (0.9-2.0) |
|  | Obesity | 11/112 | 9.8 (5.0-16.9) | 3.3 (1.0-10.2) | 1/159 | 0.6 (0.01-3.5) | 0.2 (0.02-1.6) | 66/388 | 17 (13.4-21.1) | 1.6 (1.1-2.5) |
| Any comorbidity | No | 16/251 | 6.4 (3.7-10.1) | ref. | 5/315 | 1.6 (0.5-3.7) | ref. | 123/856 | 14.4 (12.1-16.9) | ref. |
|  | Yes | 14/227 | 6.3 (3.4-10.1) | 1 (0.5-2.0) | 7/259 | 2.7 (0.1-5.5) | 1.7 (0.5-5.5) | 77/585 | 13.2 (10.5-16.2) | 0.9 (0.7-1.2) |
| Diabetes | No | 27/422 | 6.4 (4.3-9.2) | ref. | 12/510 | 2.4 (1.2-4.1) | NA | 178/1321 | 13.5 (11.7-15.4) | ref. |
|  | Yes | 3/56 | 5.4 (1.1-14.9) | 0.8 (0.2-2.8) | 0/64 | NA | NA | 22/120 | 18.3 (11.9-26.4) | 1.4 (0.9-2.3) |
| Cancer (n=2.492) | No | 30/462 | 6.5 (4.4-9.1) | NA | 11/557 | 2 (1.0-3.5) | ref. | 194/1390 | 14 (12.2-15.9) | ref. |
|  | Yes | 0/16 | NA | NA | 1/17 | 5.9 (0.1-28.6) | 3.1 (0.4-25.5) | 6/50 | 12 (4.5-24.3) | 0.8 (0.4-2.0) |
| Hypertension(n=2.491) | No | 27/377 | 7.2 (4.8-10.2) | ref. | 9/436 | 2.1 (0.9-3.9) | ref. | 168/1168 | 14.4 (12.4-16.5) | ref. |
|  | Yes | 3/101 | 3 (0.6-8.4) | 0.4 (0.1 -1.3) | 3/137 | 2.2 (0.5-6.2) | 1.1 (0.3-4.0) | 32/272 | 11.8 (8.2-16.2) | 0.8 (0.5-1.2) |
| Heart disease | No | 30/452 | 6.6 (4.5-9.3) | NA | 11/544 | 2 (1.0-3.6) | ref. | 196/1409 | 13.9 (12.1-15.8) | ref. |
|  | Yes | 0/26 | NA | NA | 1/30 | 3.3 (0.1-17.2) | 1.7 (0.2-13.4) | 4/32 | 12.5 (3.5-29.0) | 0.9 (0.3-2.5) |
| HIV/AIDS | No | 30/477 | 6.3 (4.3-8.6) | NA | 12/573 | 2.1 (0.8-4.8) | NA | 199/1431 | 13.9 (12.2-15.8) | ref. |
|  | Yes | 0/1 | NA | NA | 0/1 | NA | NA | 1/10 | 10 (0.3-44.5) | 0.7 (0.09-5.5) |
| Asthma/COPD | No | 28/438 | 6.4 (4.3-9.1) | ref. | 10/529 | 1.9 (0.9-3.4) | ref. | 187/1353 | 13.8 (12.0-15.8) | ref. |
|  | Yes | 2/40 | 5 (0.6-16.9) | 0.8 (1.2-3.4) | 2/45 | 4.4 (0.5-15.1) | 2.4 (0.5-11.4) | 13/88 | 14.8 (8.1-23.9) | 1.1 (0.6-2.0) |
| Chronic kidney disease (n=2.492) | No | 30/468 | 6.4 (4.3-9.0) | NA | 11/565 | 2 (0.9-3.5) | ref. | 197/1429 | 13.8 (12.0-15.7) | ref. |
|  | Yes | 0/10 | NA | NA | 1/9 | 11.1 (0.3-48.2) | 6.3 (0.7-54.7) | 3/11 | 27.3 (6.0-61.0) | 2.3 (0.6-8.9) |
| Endocrine system disease | No | 27/414 | 6.5 (4.3-9.3) | ref. | 12/509 | 2.4 (1.2-4.0) | NA | 183/1294 | 14.1 (12.3-16.2) | ref. |
|  | Yes | 3/64 | 4.7 (0.9-13.1) | 0.7 (0.2-2.4) | 0/65 | NA | NA | 17/147 | 11.6 (6.9-17.9) | 0.8 (0.5-1.3) |
| Osteo-articular diseases | No | 28/459 | 6.1 (4.0-8.7) | ref. | 12/549 | 2.2 (1.1-3.8) | NA | 194/1408 | 13.8 (12.0-15.7) | ref. |
|  | Yes | 2/19 | 10.5 (1.3-33.1) | 1.8 (0.4-8.2) | 0/25 | NA | NA | 6/33 | 18.2 (7.0-35.5) | 1.4 (0.6-3.4) |
| Tobacco consumption (n=2.490) | No | 25/353 | 7.1 (4.6-10.3) | ref. | 8/408 | 2 (0.9-3.8) | ref. | 154/1003 | 15.4 (13.1-17.7) | ref. |
|  | Yes | 5/125 | 4 (1.3-9.1) | 0.5 (0.2-1.5) | 4/166 | 2.4 (0.6-6.1) | 1.2 (0.4-4.2) | 44/435 | 10.1 (7.4-13.3) | 0.6 (0.4-0.9) |

*Weighted for sampling weights. OR=odds ratio. (ref)=reference category. 95%CI = 95% confidence interval.
